# The Prevalence and Predictors of Dietary Supplement Use in the Australian Population

**DOI:** 10.3390/nu9101154

**Published:** 2017-10-21

**Authors:** Stacey K. O’Brien, Eva Malacova, Jill L. Sherriff, Lucinda J. Black

**Affiliations:** School of Public Health, Curtin University, Bentley 6102, WA, Australia; stacey.k.obrien@curtin.edu.au (S.K.O.); eva.malacova@curtin.edu.au (E.M.); j.sherriff@curtin.edu.au (J.L.S.)

**Keywords:** dietary supplements, prevalence, predictors, Australian Health Survey

## Abstract

Current dietary supplement use in Australia is not well described. We investigated the prevalence and predictors of supplement use in the Australian population (*n* = 19,257) using data from the 2014–2015 National Health Survey. We reported the prevalence of supplement use by sex and age group and investigated the independent predictors of supplement use in adults, adolescents, and children using multiple logistic regression models. A total of 43.2% of adults (34.9% of males, 50.3% of females), 20.1% of adolescents (19.7% of males, 20.6% of females), and 23.5% of children (24.4% of males, 22.5% of females) used at least one dietary supplement in the previous two weeks. The most commonly used supplements were multivitamins and/or multiminerals and fish oil preparations. In adults, independent predictors of supplement use included being female, increasing age, being born outside Australia and other main English-speaking countries, having a higher education level, having a healthy BMI compared to those who were obese, being physically active, and being a non-smoker. To our knowledge, this is the first detailed investigation of dietary supplement use in a nationally-representative sample of the Australian population. Future studies investigating the contribution of supplements to overall dietary intakes of vitamins, minerals, and omega-3 fatty acids are warranted.

## 1. Introduction

Prevalence data on the regular use of dietary supplements by the general population over the last two decades are available for several countries. In the National Health and Nutrition Examination Surveys (NHANES), approximately half of the US adult population aged ≥20 years used at least one dietary supplement in the 30 days before the home interview of each survey since 1999–2000 (mean *n* per survey = 5423) [1]. This is similar to the Danish age-adjusted data (*n* = 1923) obtained by 24-h recall in those aged 35–74 years participating in the European Prospective Investigation into Cancer and Nutrition (EPIC) calibration study (1995–2000) [2], but higher than data for all other countries involved (as low as 0.5% for Greece). UK data from the National Diet and Nutrition Survey (NDNS) (September 2008–November 2010, *n* = 1491) also showed a lower prevalence of supplement use (23% for 19–64 year olds and 39% for those aged >65 years [3]) than NHANES, but this may at least partially reflect the shorter reporting period (4-day food record in the NDNS vs. 30-day recall in NHANES).

The EPIC calibration study used the same question and reporting interval across all eight countries allowing more meaningful comparisons. The wide range of prevalence rates obtained indicates that cultural and/or environmental factors influence the use of dietary supplements [2]. However, the age range of 35–74 years did not allow for the identification of prevalence rates in younger and older age groups, and age has been shown to be one of the main predictors of supplement use [4,5]. Use of dietary supplements is less prevalent in children and adolescents (e.g., 31% of those ≤19 years old, *n* = 8245 in the NHANES 2007–2010 [6]) and more prevalent in older adults [7].

The general public use dietary supplements for a variety of reasons, including as part of a healthy lifestyle, for assistance with attaining recommended intakes (e.g., calcium and vitamin D) and for management of chronic conditions (e.g., glucosamine) [4,8]. Herbal supplements are also used and concern has been raised regarding potential interactions with medications [8,9]. While the data from serial NHANES indicate that prevalence rates in the US have been constant over the last 20 years, the use of some supplements (e.g., vitamin D) has increased [1], reflecting nutrient concerns of the day. Our focus is to identify which nutrients consumed as supplements need to be included in the determination of total intake; thus, up-to-date and country-specific data on the prevalence of supplement use are required. This study aims to describe the prevalence and predictors of dietary supplement use among Australians of all ages using data from the nationally-representative 2014–2015 National Health Survey (NHS).

## 2. Materials and Methods

### 2.1. Study Population

We used questionnaire data from the NHS (*n* = 19,257), which was conducted between June 2014 and July 2015 across all States and Territories in Australia [10]. Specific methodology of the NHS can be found elsewhere [10,11]. In brief, face-to-face interviews were conducted with a randomly selected adult of the household by trained Australian Bureau of Statistics (ABS) interviewers. For child participants, a parent or guardian answered the questions on behalf of children aged <15 years [11]. Interviews were conducted in the participant’s private dwelling in metropolitan and rural areas of Australia [11]. People were excluded from the survey if they were residents of non-private dwellings, such as hotels or boarding schools, or were visitors to a selected dwelling [11]. The interview components of the NHS were conducted under the Census and Statistics Act 1905.

### 2.2. Identification of Supplement Users

In a face-to-face interview, participants were asked, “What are the names or brands of all the medications, vitamins, minerals or supplements you have taken in the last two weeks?” [10]. Participants were encouraged to have the supplements in front of them, and the name and brand were recorded by the interviewer. For the purposes of the NHS, dietary supplements refer to products defined as Complementary Medicines under the Therapeutic Goods Regulations 1990 [12]. Dietary supplements sold in Australia are regulated by the Therapeutic Goods Administration, which requires them to be listed but not registered (medicines are required to be registered) [12]. Thus, demonstration of efficacy or safety of supplements is not required. It should be noted that products available on international websites are not regulated by the Therapeutic Goods Administration [12]. The Therapeutic Goods Administration advises that consumers do not order dietary supplements over the internet unless the ingredients and legal requirements for importation into Australia are known. However, it is likely that some people obtain their dietary supplements online from international websites. The supplements recorded by the interviewer included those registered with the Therapeutic Goods Administration and those purchased overseas. The ABS categorised supplements into 28 groups (Supplementary Table S1). For the purpose of this study, any participant who reported taking at least one dietary supplement in the previous two weeks was considered a “supplement user”.

### 2.3. Potential Predictors of Supplement Use

Age was provided as a categorical variable and we re-grouped age as follows: ≤9, 10–17, 18–29, 30–49, 50–69, and ≥70 years. We further categorised these groups as adults (≥18 years, *n* = 14,560), adolescents (10–17 years, *n* = 1964) and children (≤9 years, *n* = 2733). Body mass index (BMI; measured weight in kilograms divided by measured height in metres squared) was categorized for adults according to the World Health Organization’s cut-off points for underweight, healthy weight, overweight, and obese [13]. For adolescents, cut-off points for BMI categories were assigned using half-yearly sex-and-age specific thresholds as detailed by the International Obesity Task Force [14,15]. We did not assess BMI in children, as BMI is not relevant for those aged <2 years, and our age group included children aged ≤9 years.

State/Territory was assigned for all participants as New South Wales, Victoria, Queensland, South Australia, Western Australia, Tasmania, Northern Territory, and Australian Capital Territory. Region of birth was assigned as Australia, Main English-speaking countries (Canada, Republic of Ireland, New Zealand, South Africa, United Kingdom, United States of America), and Other. As the majority of children were born in Australia and New Zealand, region of birth was assessed only in adults and adolescents.

Educational attainment for adults was defined as none after school, Certificate, Bachelor/Diploma, and postgraduate. Socioeconomic status was described by the Socio-Economic Indexes for Areas (SEIFA) 2011 Index of Relative Socio-Economic Disadvantage (IRSD). This is a general socioeconomic index that summarises a range of information about the economic and social conditions of people and households within an area with scores ranging from low (relatively greater disadvantage in general) to high (relative lack of disadvantage in general) [16]. The SEIFA IRSD was categorised into quintiles.

Physical activity for adults was defined as low, moderate, or high based on the level of physical activity over the past week, incorporating recreation, sport, transport, and fitness [11]. The data items that contributed to this variable were total minutes spent walking for transport in the last week; total minutes walked for fitness, recreation, or sport in last week; total minutes undertaken moderate exercise/physical activity in last week; total minutes undertaken vigorous exercise/physical activity in last week. Physical activity was divided into categories and each had an intensity factor score (e.g., walking for fitness = 3.5, walking for transport = 3.5, moderate exercise/physical activity = 5, and vigorous exercise/physical activity = 7.5). The intensity factor score was multiplied by the duration of physical activity. Varying levels of exercise/physical activity were defined as: low (no exercise to <800); moderate (800 to 1600, or more than 1600 but with less than 1-h vigorous physical activity); high (>1600 and with 1 h or more of vigorous physical activity). Although physical activity information was collected for all participants aged >15 years, we did not investigate physical activity in adolescents, as we did not have data for the entire adolescent group of 10–17 year olds.

Health condition was defined as whether a participant had ever had a long-term health condition, defined as a condition that had lasted, or was expected to last, for at least six months. Common long-term health conditions included asthma, arthritis, cancer, heart and circulatory conditions, diabetes mellitus, kidney disease, osteoporosis, mental or behavioural conditions, along with other less common health conditions [11]. When a participant had a past or present health condition, their health condition was defined as “yes”. For adults, smoking was defined as current smoker, past smoker, or never smoked. Self-assessed health in adults was based on how participants felt about their health and was defined as excellent, very good, good, fair, or poor.

### 2.4. Statistical Analysis

We reported the survey-weighted prevalence of dietary supplement use by sex and age group. The characteristics of the participants were reported for supplement users and non-users among adults, adolescents, and children. All prevalence data were weighted to the Australian population in 2014/2015 [11]. Survey-weighted logistic regression models were used to investigate the independent predictors of supplement use in adults (*n* = 14,560), adolescents (*n* = 1964), and children (*n* = 2733). All models were mutually adjusted for all potential predictors. Potential predictors investigated for all participants were sex, State/Territory, and socioeconomic status. For adults and adolescents, region of birth, BMI category, and health condition were also assessed. We additionally investigated age group, education, physical activity, smoking, and self-assessed health as potential predictors of supplement use in adults. The NHS is based on a stratified, multistage area sample of private households. All households were assigned analytic weights to account for their sampling probability to be included in the survey, and the models accounted for the stratification and clustering of the complex sample design using the Taylor Series Linearization method [11]. All analyses were performed using SAS version 9.4 (SAS Institute, Cary, NC, USA).

## 3. Results

**Figure 1:**
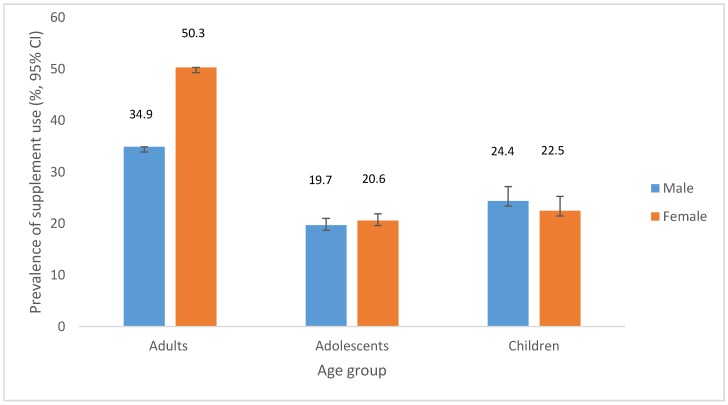
Prevalence (%, 95% CI) of supplement use in the previous two weeks for adults (aged ≥18 years, *n* = 14,560), adolescents (10–17 years, *n* = 1964), and children (≤9 years, *n* = 2733).

A total of 43.2% of adults (34.9% of males, 50.3% of females), 20.1% of adolescents (19.7% of males, 20.6% of females), and 23.5% of children (24.4% of males, 22.5% of females) used at least one dietary supplement in the previous two weeks (Figure 1). Characteristics of supplement users vs. non-users are described in Supplementary Tables S2–S4 for adults, adolescents and children, respectively. The maximum number of supplements taken by any individual was 11 among adults, five among adolescents, and seven among children. Among adults, 50.0% of supplement users took more than one supplement in the previous two weeks.

In the total population, the most commonly reported dietary supplement type was multivitamin and/or multimineral (without herbal extracts), which was used by 17.5% of the population (males 13.9%, females 20.8%) in the previous two weeks (Supplementary Table S5). A small percentage of the total population (1.5%) used multivitamin and/or multimineral with herbal extracts. Multivitamin and/or multimineral (without herbal extracts) supplement use was most commonly reported in those aged 30–49 years (22.3%; males 17.8%, females 25.8%). A total of 9.2% of the population (males 8.5%, females 9.8%) reported using fish oil preparations (without added nutrients) in the previous two weeks, most commonly in those aged 50–69 years (males 13.2%, females 16.1%).

In adults, significant independent predictors of supplement use were: being female, increasing age, being born outside Australia and other main English-speaking countries, having a higher education level, having a healthy BMI compared to those who were obese, being physically active, and being a non-smoker (Table 1). There were no significant independent predictors of supplement use in adolescents (Table 2). Adolescents in the highest quintile of socioeconomic status and having a health condition had slightly increased odds of supplement use, but the confidence intervals included 1. Being underweight had a reduced odds of supplement use, but its confidence interval also included 1. In children, residing in the Australian Capital Territory and South Australia were associated with slightly decreased odds of supplement use (Table 3). Sex and socioeconomic status were not associated with supplement use in children.

## 4. Discussion

To our knowledge, this study provides the first detailed investigation of dietary supplement use in a nationally representative sample of the Australian population. We found that for adults ≥18 years, the prevalence of supplement use was 43% (35% of males and 50% of females). Interestingly, the values are about twice those obtained in the 1995 Australian National Nutrition Survey, when 15% of adult males and 27% of females reported using dietary supplements in the previous 24 h [17]. However, apart from the 20-year time difference, this immediate reporting period (compared with two weeks in the current study) may have contributed to the lower reported prevalence of supplement use in 1995. The new Australian data are lower than the adult data of 52% (45% males, 58% females) from the NHANES (2011–2012) [1]. These surveys are comparable in that both were conducted over a similar time period, used trained interviewers [1] and referred to a recent but not immediate reporting period (30 days in NHANES vs. two weeks in the current study).

Among adults in the present study, the prevalence of supplement use increased with both age and female sex, a finding that is consistent with other studies [1,2,3,4]. The highest prevalence of supplement use was in those aged ≥70 years (40% in males and 58% in females). Australia is a multicultural country with a substantial proportion of the population born overseas in non-English speaking countries, and this analysis shows that adults born outside Australia and other main English-speaking countries are more likely to be supplement users than those born in Australia. Dietary supplement use was much less prevalent in those aged <18 years, with about a quarter of children and adolescents using supplements. These figures are lower than the third of participants aged ≤19 years identified as supplement users in the 2007–2010 NHANES [6]. Both surveys found no sex differences in the prevalence of supplement use among children and adolescents.

Multivitamins and/or multiminerals were the most commonly reported supplements across all participants in our study, which is consistent with other literature [1,5,6,8,18,19]. Fish oil preparations were the second most reported supplement in the present study, particularly in those aged ≥50 years. Prescription omega-3 preparations are also available in Australia, so total use is likely to be higher than that suggested by these data, particularly in the older age groups. The difference in adult male and female supplement use was predominantly explained by the higher use of both multivitamins and/or multiminerals and herbal products by women. While most participants reported using one dietary supplement, many preparations not referred to as multivitamins and/or multiminerals contain more than one nutrient, for example calcium and vitamin D, and fish oil with added nutrients.

Other predictors of supplement use in the Australian population, such as being a previous smoker, being physically active, and having a higher level of education, are consistent with the existing literature, as recently reviewed by Dickinson and MacKay [4]. Overall, the use of dietary supplements associates with aspects of a healthy lifestyle. Other literature investigating supplement use in infants, children and adolescents found that predictors of supplement use included the parents’ educational attainment, income and private health insurance coverage [6,18,19], which were not assessed in the current study.

Our study used data from a nationally-representative sample of the Australian population. Collecting dietary supplement use over a two-week period enabled the survey to capture episodic supplement use, typically missed in 24-h recalls [20]. Although supplement use was reported through face-to-face interviews with trained interviewers, and participants were encouraged to have the supplements in front of them, we cannot rule out errors in recording and categorising the supplements.

## 5. Conclusions

To our knowledge, this is the first detailed investigation of dietary supplement use in a nationally-representative sample of the Australian population. A substantial proportion of the Australian population reported using dietary supplements, with multivitamins and/or multiminerals being the most commonly reported type of supplement. In adults, independent predictors of supplement use included being female, increasing age, being born outside Australia and other main English-speaking countries, being physically active, higher educational attainment, having a healthy BMI compared to those who were obese, and being a non-smoker. Given that the use of multivitamins and/or multiminerals and fish oil preparations is common in the Australian population, future studies investigating the contribution of supplements to overall dietary intakes of vitamins, minerals and omega-3 fatty acids are warranted.

## Figures and Tables

**Table 1:** Adjusted logistic regression investigating independent predictors of dietary supplement use in Australian adults aged ≥18 years (*n* = 13,539).

	Adjusted OR (95% CI) ^1^
**Sex** (female vs. male)	1.94 (1.78, 2.10)
**Age group**	
18–29 years	Reference category
30–49 years	1.17 (1.02, 1.34)
50–69 years	1.61 (1.40, 1.85)
≥70 years	1.87 (1.60, 2.19)
**Region of birth**	
Australia	Reference category
Main English-speaking countries	0.96 (0.85, 1.09)
Other	1.13 (1.01, 1.26)
**State/Territory**	
New South Wales	Reference category
Victoria	1.01 (0.89, 1.14)
Queensland	1.11 (0.98, 1.25)
South Australia	1.06 (0.93, 1.21)
Western Australia	1.06 (0.92, 1.21)
Tasmania	1.08 (0.94, 1.24)
Northern Territory	0.75 (0.62, 0.91)
Australian Capital Territory	1.10 (0.95, 1.28)
**Socioeconomic status**	
Lowest quintile	Reference category
Second quintile	1.01 (0.89, 1.15)
Third quintile	1.11 (0.98, 1.27)
Fourth quintile	1.06 (0.93, 1.21)
Highest quintile	1.11 (0.97, 1.28)
**Education**	
None after school	Reference category
Certificate	1.22 (1.09, 1.37)
Bachelor/Diploma	1.46 (1.32, 1.63)
Postgraduate	1.24 (1.06, 1.45)
**BMI category**	
Healthy weight	Reference category
Underweight	1.13 (0.80, 1.60)
Overweight	0.94 (0.85, 1.04)
Obese	0.86 (0.77, 0.96)
**Physical activity**	
Low	Reference category
Moderate	1.12 (1.02, 1.24)
High	1.39 (1.21, 1.60)
**Smoking**	
Never smoked	Reference category
Past smoker	1.09 (0.99, 1.19)
Current smoker	0.72 (0.63, 0.81)
**Self-assessed health**	
Poor	Reference category
Fair	1.05 (0.85, 1.29)
Good	1.07 (0.88, 1.30)
Very good	1.05 (0.87, 1.28)
Excellent	0.98 (0.80, 1.21)
**Health condition** (yes vs. no)	1.07 (0.79, 1.44)

^1^ Adjusted for all other variables.

**Table 2:** Adjusted logistic regression investigating independent predictors of dietary supplement use in Australian adolescents aged 10–17 years (*n* = 1317).

	Adjusted OR (95% CI) ^1^
**Sex** (female vs. male)	1.12 (0.82, 1.53)
**Region of birth**	
Australia	Reference category
Main English-speaking countries	0.90 (0.44, 1.84)
Other	1.44 (0.82, 2.54)
**State/Territory**	
New South Wales	Reference category
Victoria	1.10 (0.70, 1.73)
Queensland	0.95 (0.57, 1.57)
South Australia	0.79 (0.45, 1.36)
Western Australia	1.42 (0.87, 2.31)
Tasmania	1.40 (0.83, 2.37)
Northern Territory	0.67 (0.31, 1.45)
Australian Capital Territory	1.15 (0.66, 2.00)
**Socioeconomic status**	
Lowest quintile	Reference category
Second quintile	1.12 (0.65, 1.95)
Third quintile	1.11 (0.65, 1.91)
Fourth quintile	1.13 (0.66, 1.92)
Highest quintile	1.67 (0.99, 2.82)
**BMI category**	
Healthy weight	Reference category
Underweight	0.55 (0.25, 1.22)
Overweight	0.73 (0.50, 1.06)
Obese	0.86 (0.47, 1.58)
**Health condition** (yes vs. no)	1.66 (0.84, 3.29)

^1^ Adjusted for all other variables.

**Table 3:** Adjusted logistic regression investigating independent predictors of dietary supplement use in Australian children aged ≤9 years (*n* = 2733).

	Adjusted OR (95% CI) ^1^
**Sex** (female vs. male)	0.91 (0.74, 1.21)
**State/Territory**	
New South Wales	Reference category
Victoria	0.98 (0.72, 1.32)
Queensland	0.99 (0.73, 1.34)
South Australia	0.57 (0.39, 0.83)
Western Australia	0.97 (0.70, 1.33)
Tasmania	0.78 (0.53, 1.15)
Northern Territory	0.78 (0.51, 1.19)
Australian Capital Territory	0.47 (0.32, 0.71)
**Socioeconomic status**	
Lowest quintile	Reference category
Second quintile	1.01 (0.71, 1.43)
Third quintile	0.78 (0.54, 1.12)
Fourth quintile	1.10 (0.78, 1.55)
Highest quintile	1.19 (0.85, 1.68)

^1^ Adjusted for all other variables.

## Supplementary Materials

The following are available online at www.mdpi.com/2072-6643/9/10/1154/s1, Supplementary Table S1: Supplement categories, Supplementary Table S2: Characteristics of supplement users and non-users among adults aged ≥18 years participating in the 2014–2015 National Health Survey (*n* = 14,560), Supplementary Table S3: Characteristics of supplement users and non-users among adolescents aged 10–17 years participating in the 2014–2015 National Health Survey (*n* = 1964), Supplementary Table S4: Characteristics of supplement users and non-users among children aged ≤9 years participating in the 2014–2015 National Health Survey (*n* = 2733), Supplementary Table S5: Prevalence of use of different supplement types by participants in the 2014–2015 National Health Survey (*n* = 19,257).
