# Supplementary material for: The Prevalence and Predictors of Dietary Supplement Use in the Australian Population
Source: Nutrients. 2017 Oct 21;9(10):1154. doi: 10.3390/nu9101154 (PMC5691770; doi:10.3390/nu9101154)
Supplement: Supplementary file 1 [file nutrients-09-01154-s001.pdf]

Supplementary Table S1. Supplement categories

**28 categories**	
**1**	Multivitamin and/or multimineral
**2**	Multivitamin and/or multimineral, with herbal extracts
**3**	Calcium supplements
**4**	Magnesium supplements
**5**	Zinc supplements
**6**	Other single mineral supplements
**7**	Vitamin C supplements
**8**	Vitamin E supplements
**9**	Folic acid supplements
**10**	Vitamin D supplements
**11**	Other single vitamin supplements
**12**	Fish oil supplements
**13**	Fish oil supplements with added nutrients
**14**	Cod liver oil supplements
**15**	Evening primrose oil supplements
**16**	Other lipid supplements
**17**	Herbal supplements
**18**	Herbal supplements containing caffeine
**19**	Fibre supplements
**20**	Protein or amino acid supplements
**21**	Probiotic supplements
**22**	Sports or weight control supplements
**23**	Homoeopathic supplements
**24**	Herbal medicines, topical or inhalation
**25**	Propolis or other bee product supplements
**26**	Glucosamine and/or chondroitin based
**27**	Coenzyme Q10 supplements
**28**	Other supplements

Supplementary Table S2. Characteristics of supplement users and non-users among adults aged ≥18 years participating in the 2014-2015 National Health Survey (n=14,560)

	**Supplement user**		**Supplement non-user**	
	n	**Row % (95% CI)[1]**	***n***	**Row % (95% CI)[1]**
**Sex**	***6355***	***43 (42, 44)***	***8205***	***57 (56, 58)***
Male	*2348*	35 (34, 36)	*4305*	65 (64, 66)
Female	*4007*	50 (49, 52)	*3900*	50 (48, 51)
**Age group**	***6355***		***8205***	
18-29 years	*804*	36 (34, 38)	*1460*	64 (62, 66)
30-49 years	*2221*	41 (39, 42)	*3138*	59 (58, 61)
50-69 years	*2257*	47 (45, 48)	*2515*	53 (52, 55)
≥70 years	*1073*	50 (47, 52)	*1092*	50 (48, 53)
**Region of birth**	***6355***		***8205***	
Australia	*4349*	42 (41, 44)	*5770*	58 (56, 59)
Main English-speaking countries	*780*	44 (41, 47)	*978*	56 (53, 59)
Other	*1226*	46 (43, 48)	*1457*	54 (52, 57)
**State/Territory**	***6355***		***8205***	
New South Wales	*1069*	43 (41, 45)	*1395*	57 (55, 59)
Victoria	*1097*	43 (41, 45)	*1425*	57 (55, 59)
Queensland	*1046*	44 (42, 46)	*1302*	56 (54, 58)
South Australia	*840*	43 (41, 46)	*1060*	57 (54, 59)
Western Australia	*805*	44 (42, 46)	*1023*	56 (54, 58)
Tasmania	*640*	43 (40, 45)	*842*	57 (55, 60)
Northern Territory	*263*	35 (31, 38)	*490*	65 (62, 69)
Australian Capital Territory	*595*	46 (44, 49)	*668*	54 (51, 56)
**Socioeconomic status**	***6355***		***8205***	
Lowest quintile	*1115*	41 (38, 43)	*1616*	59 (57, 62)
Second quintile	*1216*	42 (40, 44)	*1687*	58 (56, 60)
Third quintile	*1311*	44 (42, 46)	*1686*	56 (54, 58)
Fourth quintile	*1307*	44 (42, 46)	*1607*	56 (54, 58)
Highest quintile	*1406*	47 (44, 49)	*1609*	53 (51, 56)
**Education**	***6355***		***8205***	
Postgraduate	*689*	47 (44, 50)	*736*	53 (50, 56)
Bachelor/Diploma	*2029*	49 (47, 51)	*2164*	51 (49, 53)
Certificate	*1262*	41 (39, 43)	*1793*	59 (57, 61)
None after school	*2375*	40 (38, 41)	*3512*	60 (59, 62)
**BMI category**	***6355***		***8205***	
Underweight	*85*	45 (37, 53)	*118*	55 (47, 63)
Normal weight	*2251*	45 (44, 47)	*2686*	55 (53, 56)
Overweight	*2272*	43 (41, 44)	*2979*	57 (56, 59)
Obese	*1747*	41 (39, 43)	*2422*	59 (57, 61)
**Physical activity**	***6345***		***8194***	

Low	*3545*	42 (41, 43)	*4894*	58 (57, 59)
Moderate	*1931*	45 (43, 46)	*2327*	55 (54, 57)
High	*869*	47 (44, 49)	*973*	53 (51, 56)
**Smoking**	***6355***		***8205***	
Current smoker	*792*	32 (30, 35)	*1644*	68 (65, 70)
Past smoker	*2236*	46 (44, 47)	*2552*	54 (53, 56)
Never smoked	*3327*	45 (44, 46)	*4009*	55 (54, 56)
**Self-assessed health**	***6355***		***8205***	
Excellent	*1213*	44 (42, 46)	*1522*	56 (54, 58)
Very good	*2298*	44 (43, 46)	*2878*	56 (54, 57)
Good	*1797*	43 (41, 44)	*2419*	57 (56, 59)
Fair	*731*	42 (39, 45)	*961*	58 (55, 61)
Poor	*316*	42 (38, 46)	*425*	58 (54, 62)
**Health condition**	***6028***		***7531***	
Yes	*5933*	44 (43, 45)	*7354*	56 (55, 57)
No	*95*	38 (31, 45)	*177*	62 (55, 69)

[1]Percentages weighted to the Australian population in 2014/2015

Supplementary Table S3. Characteristics of supplement users and non-users among adolescents aged 10-17 years participating in the 2014-2015 National Health Survey (n=1,964)

	Supplement user		Supplement non-user	
	n	**Row % (95% CI)[1]**	***n***	**Row % (95% CI)[1]**
**Sex**	***410***	***20 (18, 22)***	***1554***	***80 (78, 82)***
Male	*212*	20 (17, 23)	*806*	80 (77, 83)
Female	*198*	21 (18, 24)	*748*	79 (76, 82)
**Region of birth**	***410***		***1554***	
Australia	*351*	20 (18, 22)	*1364*	80 (78, 82)
Main English-speaking countries	*24*	23 (14, 32)	*75*	77 (68, 86)
Other	*35*	21 (14, 29)	*115*	79 (71, 86)
**State/Territory**	***410***		***1554***	
New South Wales	*66*	19 (15, 23)	*266*	81 (77, 85)
Victoria	*75*	21 (16, 25)	*284*	79 (75, 84)
Queensland	*56*	18 (14, 22)	*248*	82 (78, 86)
South Australia	*39*	17 (12, 21)	*187*	83 (79, 88)
Western Australia	*67*	27 (21, 32)	*186*	73 (68, 79)
Tasmania	*5*	25 (19, 32)	*150*	75 (68, 81)
Northern Territory	*16*	12 (6, 18)	*105*	88 (82, 94)
Australian Capital Territory	*40*	23 (17, 30)	*128*	77 (70, 83)
**Socioeconomic status**	***410***		***1554***	
Lowest quintile	*49*	16 (11, 21)	*248*	84 (79, 89)
Second quintile	*73*	19 (15, 24)	*269*	81 (76, 85)
Third quintile	*83*	19 (14, 23)	*336*	81 (77, 86)
Fourth quintile	*95*	21 (17, 26)	*346*	79 (74, 83)
Highest quintile	*110*	24 (19, 29)	*355*	76 (71, 81)
**BMI category**	***410***		***1554***	
Underweight	*19*	17 (9, 25)	*93*	83 (75, 91)
Normal weight	*271*	21 (19, 24)	*989*	79 (76, 81)
Overweight	*89*	16 (13, 20)	*358*	84 (80, 87)
Obese	*31*	23 (15, 31)	*114*	77 (69, 85)
**Health condition**	***305***		***1012***	
Yes	*288*	23 (20, 26)	*994*	77 (74, 80)
No	*17*	15 (6, 23)	*68*	85 (77, 94)

[1]Percentages weighted to the Australian population in 2014/2015

Supplementary Table S4. Characteristics of supplement users and non-users among children aged ≤9 years participating in the 2014-2015 National Health Survey (n=2,733)

	**Supplement user**		**Supplement non-user**	
	n	**Row % (95% CI)**[1]	***n***	**Row % (95% CI)**[1]
**Sex**	***608***	***24 (22, 25)***	***2125***	***76 (75, 78)***
Male	*321*	24 (22, 27)	*1116*	76 (73, 78)
Female	*287*	23 (20, 25)	*1009*	77 (75, 80)
**State/Territory**	***608***		***2125***	
New South Wales	*126*	25 (21, 29)	*355*	75 (71, 79)
Victoria	*112*	24 (20, 28)	*342*	76 (72, 80)
Queensland	*104*	24 (20, 28)	*323*	76 (72, 80)
South Australia	*48*	15 (11, 19)	*251*	85 (81, 89)
Western Australia	*90*	24 (20, 29)	*282*	76 (71, 80)
Tasmania	*48*	20 (15, 25)	*186*	80 (75, 85)
Northern Territory	*39*	20 (14, 26)	*148*	80 (74, 86)
Australian Capital Territory	*41*	14 (10, 19)	*238*	86 (81, 90)
**Socioeconomic status**	***608***		***2125***	
Lowest quintile	*96*	23 (19, 28)	*353*	77 (72, 81)
Second quintile	*124*	23 (19, 28)	*410*	77 (72, 81)
Third quintile	*99*	19 (15, 23)	*431*	81 (77, 85)
Fourth quintile	*138*	25 (21, 29)	*427*	75 (71, 79)
Highest quintile	*151*	26 (22, 31)	*504*	74 (69, 78)

[1]Percentages weighted to the Australian population in 2014/2015

Supplementary Table S5. Prevalence of use of different supplement types by participants in the 2014-2015 National Health Survey (n=19,257)

	Age (years)						
n	**≤9**	**10-17**	**18-29**	**30-49**	**50-69**	**≥70**	**All**
			Total				
	2733	*1964*	*2264*	*5359*	*4772*	*2165*	*19257*
	% (95% CI)[1]	% (95% CI)[1]	% (95% CI)[1]	% (95% CI)[1]	% (95% CI)[1]	% (95% CI)[1]	% (95% CI)[1]
All supplements	23.5 (21.7, 25.4)	20.1 (18.1, 22.2)	36.1 (33.8, 38.5)	40.5 (39.0, 42.1)	46.8 (45.1, 48.4)	49.5 (47.1, 51.9)	38.0 (37.2, 38.8)
Multivitamin/mineral	12.4 (10.9, 13.9)	10.1 (8.5, 11.6)	19.0 (17.1, 20.8)	22.3 (21.0, 23.6)	18.3 (17.0, 19.5)	16.7 (14.9, 18.5)	17.5 (16.9, 18.2)
Multivitamin/mineral, with herbal extracts	0.6 (0.2, 0.9)	0.7 (0.3, 1.1)	1.9 (1.2, 2.6)	1.7 (1.3, 2.1)	1.8 (1.3, 2.2)	1.7 (1.0, 2.3)	1.5 (1.3, 1.7)
Calcium	0.6 (0.2, 0.9)	0.2 (0.02, 0.4)	1.3 (0.8, 1.9)	2.5 (2.0, 3.0)	6.2 (5.4, 6.9)	9.6 (8.2, 11.0)	3.6 (3.3, 3.9)
Magnesium	0.3 (0.03, 0.5)	0.6 (0.2, 1.0)	2.3 (1.6, 3.0)	3.3 (2.8, 4.0)	6.0 (5.2, 6.7)	6.9 (5.6, 8.1)	3.6 (3.3, 3.9)
Zinc	0.2 (0.000, 0.4)	0.2 (0.000, 0.4)	0.6 (0.2, 1.0)	0.4 (0.2, 0.5)	0.5 (0.3, 0.8)	0.2 (0.003, 0.4)	0.4 (0.3, 0.5)
Other single mineral	0.1 (0.000, 0.2)	0.1 (0.000, 0.1)	0.8 (0.3, 1.3)	0.5 (0.3, 0.7)	1.3 (0.9, 1.6)	1.8 (1.1, 2.5)	0.8 (0.6, 0.9)
Vitamin C	3.2 (2.4, 3.9)	5.0 (3.9, 6.2)	6.5 (5.3, 7.7)	5.2 (4.5, 5.9)	5.1 (4.4, 5.8)	3.7 (2.8, 4.6)	4.9 (4.5, 5.2)
Vitamin E	0.1 (0.000, 0.2)	-	0.1 (0.000, 0.3)	0.4 (0.2, 0.6)	1.1 (0.8, 1.5)	1.3 (0.8, 1.9)	0.6 (0.4, 0.7)
Folic acid	-	-	0.7 (0.3, 1.1)	0.6 (0.4, 0.8)	0.4 (0.2, 0.6)	0.7 (0.3, 1.1)	0.4 (0.3, 0.5)
Vitamin D	1.3 (0.8, 1.8)	1.3 (0.7, 1.9)	3.4 (2.6, 4.3)	6.3 (5.5, 7.0)	11.2 (10.2, 12.2)	16.2 (14.5, 18.0)	7.1 (6.6, 7.5)
Other single vitamin	0.2 (0.002, 0.4)	0.3 (0.1, 0.6)	0.9 (0.5, 1.4)	1.6 (1.2, 2.0)	2.3 (1.8, 2.8)	2.1 (1.4, 2.8)	1.4 (1.2, 1.6)
Fish oil	5.2 (4.2, 6.2)	4.1 (3.1, 5.1)	6.0 (4.9, 7.2)	7.2 (6.5, 8.0)	14.8 (13.6, 15.9)	14.3 (12.6, 16.0)	9.2 (8.7, 9.6)

Fish oil with added nutrients	1.2 (0.7, 1.7)	0.6 (0.2, 1.0)	0.8 (0.4, 1.2)	0.9 (0.6, 1.2)	0.5 (0.3, 0.8)	0.7 (0.3, 1.1)	0.8 (0.6, 0.9)
Cod liver oil	0.1 (0.000, 0.3)	0.1 (0.000, 0.2)	0.1 (0.000, 0.3)	0.3 (0.1, 0.5)	0.3 (0.2, 0.5)	0.5 (0.2, 0.8)	0.3 (0.2, 0.4)
Evening primrose oil	-	0.3 (0.03, 0.5)	0.3 (0.03, 0.6)	0.7 (0.4, 0.9)	0.6 (0.4, 0.9)	0.1 (0.000, 0.3)	0.4 (0.3, 0.5)
Other lipid	0.1 (0.000, 0.2)	0.3 (0.05, 0.6)	0.8 (0.4, 1.2)	1.2 (0.9, 1.6)	3.7 (3.1, 4.3)	2.6 (1.9, 3.4)	1.7 (1.5, 1.9)
Herbal	1.4 (0.8, 1.9)	2.3 (1.5, 3.0)	4.7 (3.7, 5.7)	7.0 (6.2, 7.8)	8.4 (7.5, 9.3)	7.0 (5.8, 8.3)	5.8 (5.4, 6.2)
Herbal containing caffeine	-	-	0.1 (0.000, 0.2)	0.1 (0.04, 0.2)	0.1 (0.01, 0.2)	0.1 (0.000, 0.3)	0.1 (0.04, 0.1)
Homeopathic	0.6 (0.3, 0.9)	0.1 (0.000, 0.2)	0.3 (0.02, 0.5)	0.2 (0.1, 0.3)	0.1 (0.03, 0.2)	0.04 (0.000, 0.1)	0.2 (0.1, 0.3)
Herbal medicines, topical or inhalation	0.6 (0.2, 0.9)	0.1 (0.000, 0.2)	0.3 (0.1, 0.6)	0.2 (0.1, 0.4)	0.5 (0.3, 0.7)	0.2 (0.000, 0.4)	0.3 (0.2, 0.4)
Fibre	0.1 (0.000, 0.2)	0.2 (0.000, 0.4)	0.3 (0.04, 0.5)	0.5 (0.3, 0.7)	0.6 (0.3, 0.8)	0.4 (0.1, 0.8)	0.4 (0.3, 0.5)
Protein or amino acid	0.2 (0.000, 0.4)	0.4 (0.1, 0.7)	2.0 (1.4, 2.7)	1.5 (1.1, 1.9)	0.6 (0.3, 0.8)	0.3 (0.03, 0.5)	0.9 (0.7, 1.1)
Sports or weight control	0.1 (0.000, 0.2)	0.3 (0.000, 0.6)	2.0 (1.3, 2.6)	0.9 (0.6, 1.2)	0.3 (0.1, 0.4)	0.2 (0.000, 0.3)	0.6 (0.5, 0.7)
Probiotic	2.9 (2.1, 3.6)	0.9 (0.4, 1.4)	2.4 (1.6, 3.1)	2.5 (2.0, 3.0)	2.1 (1.7, 2.6)	1.6 (1.0, 2.2)	2.2 (1.9, 2.4)
Propolis or other bee product	0.2 (0.000, 0.5)	-	0.1 (0.000, 0.2)	0.2 (0.1, 0.4)	0.3 (0.1, 0.5)	0.3 (0.000, 0.5)	0.2 (0.1, 0.3)
Glucosamine and/or chondroitin	0.01 (0.000, 0.02)	0.1 (0.000, 0.3)	0.7 (0.3, 1.1)	2.0 (1.6, 2.4)	7.4 (6.6, 8.3)	8.1 (6.8, 9.4)	3.4 (3.1, 3.7)
Coenzyme Q10	-	-	0.4 (0.1, 0.6)	0.4 (0.2, 0.6)	1.7 (1.3, 2.2)	2.0 (1.3, 2.7)	0.8 (0.7, 1.0)
Other	0.3 (0.000, 0.4)	0.1 (0.000, 0.3)	0.2 (0.05, 0.4)	0.5 (0.3, 0.8)	0.9 (0.6, 1.2)	1.0 (0.5, 1.4)	0.6 (0.4, 0.7)

Males

n	*1437*	*1018*	*1092*	*2387*	*2216*	*958*	*9108*
	% (95% CI)	% (95% CI)	% (95% CI)	% (95% CI)	% (95% CI)	% (95% CI)	% (95% CI)
All supplements	24.4 (21.8, 27.1)	19.7 (16.8, 22.5)	29.3 (26.2, 32.4)	33.3 (31.1, 35.5)	37.2 (34.8, 39.5)	39.7 (36.1, 43.3)	31.4 (30.3, 32.6)
Multivitamin/mineral	13.2 (11.1, 15.3)	9.3 (7.3, 11.3)	14.5 (12.1, 16.8)	17.8 (16.0, 19.5)	13.4 (11.8, 15.0)	11.9 (9.5, 14.3)	13.9 (13.1, 14.7)
Multivitamin/mineral, with herbal extracts	0.5 (0.1, 1.0)	0.5 (0.01, 1.1)	1.2 (0.4, 2.0)	0.8 (0.4, 1.2)	1.1 (0.6, 1.5)	1.1 (0.5, 1.7)	0.9 (0.7, 1.1)
Calcium	0.6 (0.1, 1.1)	0.2 (0.000, 0.4)	1.3 (0.4, 2.1)	1.4 (0.8, 2.0)	2.6 (1.8, 3.4)	5.4 (3.6, 7.1)	1.8 (1.5, 2.2)
Magnesium	0.3 (0.000, 0.7)	0.6 (0.04, 1.1)	1.7 (0.8, 2.6)	2.4 (1.7, 3.2)	4.1 (3.1, 5.1)	5.4 (3.7, 7.1)	2.5 (2.1, 2.9)
Zinc	0.1 (0.000, 0.4)	0.1 (0.000, 0.3)	0.6 (0.1, 1.1)	0.4 (0.1, 0.7)	0.5 (0.1, 0.9)	0.2 (0.000, 0.5)	0.3 (0.2, 0.5)
Other single mineral	0.1 (0.000, 0.3)	0.1 (0.000, 0.2)	0.3 (0.000, 0.6)	0.1 (0.000, 0.2)	0.9 (0.4, 1.3)	1.4 (0.5, 2.4)	0.5 (0.3, 0.6)
Vitamin C	3.6 (2.4, 4.7)	5.2 (3.6, 6.9)	6.3 (4.7, 8.0)	5.1 (4.0, 6.1)	4.2 (3.2, 5.2)	3.4 (2.0, 4.7)	4.6 (4.1, 5.1)
Vitamin E	0.1 (0.000, 0.3)	-	0.2 (0.000, 0.4)	0.2 (0.01, 0.5)	1.0 (0.5, 1.5)	0.8 (0.1, 1.5)	0.4 (0.3, 0.6)
Folic acid	-	-	0.2 (0.000, 0.5)	0.1 (0.000, 0.2)	0.2 (0.003, 0.5)	0.5 (0.02, 1.0)	0.2 (0.1, 0.3)
Vitamin D	1.4 (0.6, 2.1)	0.4 (0.03, 0.7)	1.8 (0.9, 2.7)	4.0 (3.1, 4.9)	5.5 (4.4, 6.6)	9.3 (7.3, 11.4)	3.8 (3.4, 4.3)
Other single vitamin	0.1 (0.000, 0.3)	0.2 (0.000, 0.3)	1.0 (0.4, 1.6)	0.8 (0.4, 1.2)	1.7 (1.1, 2.4)	2.4 (1.2, 3.5)	1.0 (0.8, 1.3)
Fish oil	6.4 (4.9, 7.9)	5.0 (3.5, 6.5)	5.9 (4.3, 7.5)	7.0 (5.8, 8.1)	13.2 (11.6, 14.8)	11.0 (8.8, 13.2)	8.5 (7.8, 9.2)
Fish oil with added nutrients	0.9 (0.3, 1.5)	0.5 (0.02, 1.0)	0.02 (0.000, 0.05)	0.2 (0.01, 0.4)	0.4 (0.1, 0.7)	0.8 (0.2, 1.4)	0.4 (0.3, 0.6)
Cod liver oil	0.3 (0.000, 0.6)	0.1 (0.000, 0.3)	-	0.1 (0.000, 0.3)	0.2 (0.02, 0.4)	0.2 (0.000, 0.4)	0.2 (0.1, 0.2)
Evening primrose oil	-	0.3	0.1	0.005	0.2	0.06	0.1

		(0.000, 0.6)	(0.000, 0.2)	(0.000, 0.01)	(0.01, 0.4)	(0.000, 0.2)	(0.03, 0.2)
Other lipid	0.2	0.4	0.9	1.2	2.5	2.2	1.3
	(0.000, 0.5)	(0.000, 0.9)	(0.3, 1.6)	(0.7, 1.6)	(1.8, 3.3)	(1.1, 3.3)	(1.1, 1.6)
Herbal	1.4	1.6	3.4	4.7	5.2	6.7	4.0
	(0.7, 2.1)	(0.8, 2.5)	(2.2, 4.5)	(3.7, 5.6)	(4.1, 6.3)	(4.8, 8.5)	(3.5, 4.4)
Herbal containing caffeine	-	-	0.1	0.2	0.1	-	0.1
			(0.000, 0.3)	(0.000, 0.3)	(0.000, 0.3)		(0.02, 0.2)
Homeopathic	0.5	0.1	0.1	0.1	0.03	-	0.1
	(0.1, 1.0)	(0.000, 0.3)	(0.000, 0.4)	(0.000, 0.2)	(0.000, 0.1)		(0.1, 0.2)
Herbal medicines, topical or inhalation	0.8	0.2	0.4	0.1	0.3	0.1	0.3
	(0.2, 1.4)	(0.000, 0.5)	(0.000, 0.8)	(0.000, 0.2)	(0.1, 0.6)	(0.000, 0.3)	(0.2, 0.4)
Fibre	0.1	0.3	0.4	0.4	0.4	-	0.3
	(0.000, 0.2)	(0.000, 0.8)	(0.000, 0.8)	(0.1, 0.7)	(0.1, 0.7)		(0.2, 0.4)
Protein or amino acid	0.2	0.7	2.9	1.7	0.5	0.3	1.0
	(0.000, 0.5)	(0.1, 1.2)	(1.7, 4.0)	(1.1, 2.2)	(0.1, 0.9)	(0.000, 0.6)	(0.8, 1.3)
Sports or weight control	0.1	0.6	3.1	1.3	0.3	0.1	0.9
	(0.000, 0.3)	(0.000, 1.2)	(1.9, 4.2)	(0.8, 1.8)	(0.03, 0.5)	(0.000, 0.3)	(0.7, 1.1)
Probiotic	2.7	0.9	1.6	1.1	0.8	1.6	1.4
	(1.7, 3.7)	(0.3, 1.6)	(0.7, 2.5)	(0.7, 1.6)	(0.4, 1.3)	(0.6, 2.6)	(1.1, 1.6)
Propolis or other bee product	0.4	-	-	0.2	0.3	0.3	0.2
	(0.000, 0.9)			(0.000, 0.4)	(0.000, 0.5)	(0.000, 0.8)	(0.1, 0.3)
Glucosamine and/or chondroitin	0.01	0.2	0.7	2.5	6.1	6.4	2.9
	(0.000, 0.04)	(0.000, 0.5)	(0.1, 1.2)	(1.8, 3.2)	(5.0, 7.3)	(4.6, 8.2)	(2.5, 3.3)
Coenzyme Q10	-	-	0.1	0.4	1.5	1.7	0.7
			(0.000, 0.2)	(0.1, 0.8)	(0.9, 2.1)	(0.8, 2.6)	(0.5, 0.9)
Other	-	-	0.2	0.5	0.6	1.3	0.4
			(0.000, 0.4)	(0.1, 0.8)	(0.2, 0.9)	(0.5, 2.0)	(0.3, 0.6)
			Females				
n	*1296*	*946*	*1172*	*2972*	*2556*	*1207*	*10149*
	%	%	%	%	%	%	%
	(95% CI)	(95% CI)	(95% CI)	(95% CI)	(95% CI)	(95% CI)	(95% CI)
All supplements	22.5	20.6	42.5	46.1	55.2	57.5	43.9
	(19.9, 25.2)	(17.6, 23.6)	(39.2, 45.9)	(44.0, 48.2)	(53.0, 57.5)	(54.3, 60.7)	(42.7, 45.1)

Multivitamin/mineral	11.5 (9.5, 13.5)	10.9 (8.5, 13.2)	23.2 (20.3, 26.0)	25.8 (24.0, 27.6)	22.6 (20.7, 24.4)	20.6 (18.0, 23.2)	20.8 (19.9, 21.7)
Multivitamin/mineral, with herbal extracts	0.6 (0.2, 1.0)	0.9 (0.1, 1.6)	2.6 (1.5, 3.7)	2.4 (1.8, 3.0)	2.4 (1.7, 3.1)	2.1 (1.1, 3.1)	2.0 (1.7, 2.3)
Calcium	0.6 (0.1, 1.1)	0.2 (0.000, 0.5)	1.4 (0.7, 2.2)	3.4 (2.6, 4.1)	9.3 (8.0, 10.6)	13.0 (10.9, 15.2)	5.1 (4.6, 5.6)
Magnesium	0.3 (0.000, 0.6)	0.6 (0.1, 1.2)	2.9 (1.8, 4.0)	4.1 (3.3, 5.0)	7.6 (6.4, 8.7)	8.1 (6.3, 9.8)	4.5 (4.0, 5.0)
Zinc	0.2 (0.000, 0.5)	0.3 (0.000, 0.7)	0.6 (0.03, 1.2)	0.3 (0.1, 0.6)	0.5 (0.2, 0.8)	0.2 (0.000, 0.5)	0.4 (0.2, 0.5)
Other single mineral	0.03 (0.000, 0.1)	0.1 (0.000, 0.2)	1.3 (0.5, 2.1)	0.8 (0.4, 1.1)	1.6 (1.0, 2.2)	2.1 (1.2, 3.1)	1.1 (0.8, 1.3)
Vitamin C	2.7 (1.7, 3.7)	4.8 (3.2, 6.4)	6.7 (5.0, 8.4)	5.3 (4.3, 6.2)	5.9 (4.8, 6.9)	4.0 (2.7, 5.3)	5.1 (4.6, 5.6)
Vitamin E	0.1 (0.000, 0.2)	-	0.1 (0.000, 0.2)	0.5 (0.2, 0.8)	1.3 (0.8, 1.8)	1.8 (0.9, 2.7)	0.7 (0.5, 0.9)
Folic acid	-	-	1.2 (0.4, 2.0)	1.0 (0.6, 1.4)	0.5 (0.2, 0.8)	0.8 (0.2, 1.4)	0.7 (0.5, 0.8)
Vitamin D	1.2 (0.5, 1.9)	2.2 (1.1, 3.3)	5.0 (3.5, 6.5)	8.0 (6.9, 9.1)	16.2 (14.6, 17.9)	21.8 (19.2, 24.5)	10.0 (9.3, 10.7)
Other single vitamin	0.4 (0.000, 0.8)	0.6 (0.002, 1.1)	0.9 (0.3, 1.5)	2.2 (1.5, 2.8)	2.8 (2.0, 3.6)	1.9 (1.0, 2.7)	1.8 (1.4, 2.1)
Fish oil	3.9 (2.7, 5.1)	3.2 (1.9, 4.4)	6.2 (4.6, 7.8)	7.5 (6.4, 8.5)	16.1 (14.5, 17.7)	17.0 (14.5, 19.4)	9.8 (9.1, 10.4)
Fish oil with added nutrients	1.5 (0.7, 2.3)	0.7 (0.1, 1.3)	1.4 (0.7, 2.2)	1.5 (1.0, 2.0)	0.6 (0.2, 1.0)	0.7 (0.2, 1.2)	1.1 (0.8, 1.3)
Cod liver oil	-	-	0.2 (0.000, 0.6)	0.5 (0.2, 0.7)	0.5 (0.1, 0.8)	0.7 (0.2, 1.3)	0.4 (0.2, 0.5)
Evening primrose oil	-	0.3 (0.01, 0.5)	0.5 (0.01, 1.0)	1.2 (0.8, 1.7)	1.0 (0.6, 1.4)	0.2 (0.000, 0.4)	0.7 (0.5, 0.9)
Other lipid	-	0.3 (0.000, 0.6)	0.6 (0.1, 1.1)	1.3 (0.8, 1.7)	4.7 (3.8, 5.7)	3.0 (1.9, 4.0)	2.0 (1.7, 2.3)
Herbal	1.3 (0.6, 2.1)	2.9 (1.6, 4.2)	5.9 (4.4, 7.5)	8.7 (7.6, 9.9)	11.2 (9.8, 12.7)	7.3 (5.7, 9.0)	7.4 (6.8, 8.0)

Herbal containing caffeine	-	-	0.02 (0.000, 0.05)	0.1 (0.004, 0.2)	0.1 (0.000, 0.2)	0.2 (0.000, 0.5)	0.1 (0.03, 0.1)
Homeopathic	0.6 (0.2, 1.0)	-	0.4 (0.000, 0.9)	0.3 (0.1, 0.4)	0.2 (0.03, 0.3)	0.1 (0.000, 0.2)	0.2 (0.1, 0.3)
Herbal medicines, topical or inhalation	0.3 (0.000, 0.7)	-	0.3 (0.000, 0.7)	0.4 (0.1, 0.6)	0.6 (0.3, 1.0)	0.3 (0.000, 0.6)	0.4 (0.2, 0.5)
Fibre	0.1 (0.000, 0.3)	-	0.2 (0.000, 0.5)	0.6 (0.3, 0.9)	0.8 (0.3, 1.2)	0.8 (0.2, 1.4)	0.5 (0.3, 0.7)
Protein or amino acid	0.1 (0.000, 0.4)	0.03 (0.000, 0.1)	1.2 (0.5, 2.0)	1.4 (0.9, 1.9)	0.7 (0.3, 1.0)	0.3 (0.000, 0.6)	0.8 (0.6, 1.0)
Sports or weight control	-	-	1.0 (0.3, 1.6)	0.5 (0.2, 0.8)	0.3 (0.1, 0.5)	0.2 (0.000, 0.4)	0.4 (0.2, 0.5)
Probiotic	3.1 (2.0, 4.1)	0.9 (0.2, 1.5)	3.1 (1.9, 4.3)	3.6 (2.8, 4.4)	3.3 (2.5, 4.0)	1.6 (0.8, 2.3)	2.9 (2.5, 3.2)
Propolis or other bee product	-	-	0.1 (0.000, 0.3)	0.3 (0.002, 0.6)	0.3 (0.1, 0.6)	0.2 (0.000, 0.5)	0.2 (0.1, 0.3)
Glucosamine and/or chondroitin	-	-	0.7 (0.2, 1.3)	1.6 (1.1, 2.1)	8.6 (7.3, 9.8)	9.4 (7.5, 11.3)	3.8 (3.4, 4.3)
Coenzyme Q10	-	-	0.6 (0.1, 1.1)	0.4 (0.1, 0.7)	1.9 (1.3, 2.6)	2.2 (1.2, 3.3)	1.0 (0.7, 1.2)
Other	0.5 (0.000, 1.0)	0.2 (0.000, 0.6)	0.2 (0.000, 0.5)	0.6 (0.3, 0.9)	1.2 (0.7, 1.7)	0.8 (0.2, 1.3)	0.7 (0.5, 0.9)

[1]Percentages weighted to the Australian population in 2014/2015
